# Effects of Vehicle Speed on Flight Initiation by Turkey Vultures: Implications for Bird-Vehicle Collisions

**DOI:** 10.1371/journal.pone.0087944

**Published:** 2014-02-04

**Authors:** Travis L. DeVault, Bradley F. Blackwell, Thomas W. Seamans, Steven L. Lima, Esteban Fernández-Juricic

**Affiliations:** 1 United States Department of Agriculture, Animal and Plant Health Inspection Service, Wildlife Services, National Wildlife Research Center, Sandusky, Ohio, United States of America; 2 Department of Biology, Indiana State University, Terre Haute, Indiana, United States of America; 3 Department of Biological Sciences, Purdue University, West Lafayette, Indiana, United States of America; University of Lethbridge, Canada

## Abstract

The avoidance of motorized vehicles is a common challenge for birds in the modern world. Birds appear to rely on antipredator behaviors to avoid vehicles, but modern vehicles (automobiles and aircraft) are faster than natural predators. Thus, birds may be relatively ill-equipped, in terms of sensory capabilities and behaviors, to avoid vehicles. We examined the idea that birds may be unable to accurately assess particularly high speeds of approaching vehicles, which could contribute to miscalculations in avoidance behaviors and ultimately cause collisions. We baited turkey vultures (*Cathartes aura*) to roads with animal carcasses and measured flight initiation distance and effective time-to-collision in response to a truck driving directly towards vultures from a starting distance of 1.13 km and at one of three speeds: 30, 60, or 90 kph (no vultures were struck). Flight initiation distance of vultures increased by a factor of 1.85 as speed increased from 30 to 90 kph. However, for 90-kph approaches there was no clear trend in flight initiation distance across replicates: birds appeared equally likely to initiate escape behavior at 40 m as at 220 m. Time-to-collision decreased by a factor of 0.62 with approach speeds from 30 to 90 kph. Also, at 90 kph, four vehicle approaches (17%) resulted in near collisions with vultures (time-to-collision ≤1.7 s), compared to none during 60 kph approaches and one during 30 kph approaches (4%). Our findings suggest that antipredator behaviors in turkey vultures, particularly stimulus processing and response, might not be well tuned to vehicles approaching at speeds ≥90 kph. The possible inability of turkey vultures to react appropriately to high-speed vehicles could be common among birds, and might represent an important determinant of bird-vehicle collisions.

## Introduction

The avoidance of motorized vehicles is a common challenge for birds in the modern world. Although interactions between birds and vehicles usually result in successful avoidance, bird fatalities from collisions with automobiles [1], [2] and aircraft [3] occur frequently worldwide. Erickson et al. [4] estimated that there are 80 million bird fatalities annually from automobile collisions in the USA alone, and the actual number of these fatal collisions could be much higher (possibly from 2 to 39 times that number) given the substantial biases associated with investigators finding carcasses and scavenger removal along roads [5], [6]. Although many bird populations are robust enough to withstand losses from automobile collisions without suffering major declines, automobile collisions can be important causes of mortality for rare and endangered species such as Florida scrub-jays (*Aphelocoma coerulescens*) [2], [7]–[9]. Bird collisions with aircraft occur much less frequently than collisions with automobiles; for example, about 10,000 bird collisions with US civil aircraft are reported annually to the Federal Aviation Administration under a voluntary reporting system [3]. Nevertheless, these collisions are a serious threat to aviation safety [10].

Mitigation strategies for reducing bird-vehicle collisions (BVCs) on roads are usually local and often involve habitat manipulations (e.g., crossing structures, fences to encourage higher-altitude flights across roads, removal of roadside fruiting trees and road-killed animals) [11]. Efforts to reduce BVCs with aircraft are also concentrated locally (i.e., on airport properties) and rely heavily on habitat manipulations and nonlethal dispersal using pyrotechnics and other deterrents [10], [12], [13]. Unfortunately, the effectiveness of strategies to mitigate BVCs on roads is largely unknown [14], and despite demonstrated success in mitigating on-airport BVCs, damaging collisions away from the airport (i.e., at higher altitudes) continue to increase [15].

A better understanding of how birds perceive and react to vehicles would aid the development of new strategies to reduce BVCs [16]–[19]. Unfortunately, very little is understood about the fundamental causes of BVCs. What goes wrong, from the bird’s point of view, when collisions occur? Why are some birds unable to avoid large, noisy vehicles travelling along a predictable path? For a bird (or any other animal) to avoid a vehicle on a collision course, it must successfully detect the object, assess it as a threat, and initiate an appropriate evasion response—failure at any of these steps can result in a collision (unpublished data). Thus, there are sensory, cognitive, and behavioral hurdles that must be overcome to successfully avoid an oncoming vehicle.

Modern vehicles are usually much faster than predators, and have been common in industrialized nations for only about 100 years—possibly not long enough for birds to have developed specialized avoidance mechanisms [7], [20], [21]. Instead, it appears that birds generally rely on antipredator behaviors [22] to avoid vehicles [19], [23], [24]. Birds may therefore use simple rules governing antipredator behavior in response to vehicle approach, such as those based on object size, speed and direction of approach, or a spatial margin of safety [25]–[28]. However, because of the nature of visual information of oncoming objects, particularly the near-exponential growth of the angle subtended by the object on the retina as it approaches the observer (i.e., the looming response [29], [30]), it seems plausible that unnaturally fast vehicles might overwhelm sensory and brain-processing mechanisms normally used to avoid predators. Such errors in assessing the speed of oncoming vehicles, if present, could contribute to ineffective avoidance responses and result in BVCs [17], [31].

Although several authors have speculated about the importance of vehicle speed in contributing to animal-vehicle collisions [1], [31], the topic has been largely unexplored. One recent study found that several species of European birds adjusted their flight initiation distance (FID) [26], [32] according to the posted speed limit, but not the actual speed of the oncoming vehicle [33]. This suggests that birds are able to associate some road sections with average vehicle speed, although the role of proximate risk assessment (i.e., for individual oncoming vehicles) remains unclear.

In this study we used an experimental approach to investigate the effects of vehicle speed on birds in a straightforward but unique way: we drove a vehicle directly towards turkey vultures (*Cathartes aura*) baited to the middle of roads at typical vehicle speeds (30–90 kph) and measured their reactions. Turkey vultures are common scavengers throughout much of North and South America that often feed along roads [34] and regularly cause damaging aircraft strikes [13], [35]. We determined how vehicle speed influenced the initiation of vehicle avoidance behaviors by characterizing the measured responses of vultures in spatial (FID) and temporal (time-to-collisions; TTC) terms. Such information could help elucidate some fundamental mechanisms involved in BVCs and thus inform current management practices and new approaches to enhance avian responses in the future. Given the evolutionary novelty of high-speed vehicles (i.e., likely imposing challenges for cognitive processing and subsequent behavioral responses) and the frequency of BVCs observed worldwide, we predicted that vultures would react to the approaching vehicle with less time to spare with increasing vehicle speed.

## Materials and Methods

### Study Area

We conducted our study at the National Aeronautic and Space Administration’s Plum Brook Station, Erie County, Ohio, United States of America (41220N, 82410W). The 2200-ha Plum Brook Station is enclosed by a high fence and has limited public access. Habitats consist of canopy-dogwood (*Cornus* spp.; 39%), old field and grasslands (31%), open woodlands (15%), and mixed hardwood forests (11%) interspersed by buildings and other structures. Numerous paved roads are located on the facility, where the terrain is generally flat. The number of turkey vultures that regularly forage at Plum Brook Station is unknown, although they are abundant during the breeding season. There are several long-term nocturnal roosts within 10 km of Plum Brook Station, one of which regularly contains >100 turkey vultures during summer (unpublished data). All of these vultures are likely familiar with vehicle traffic.

### Field Methods

We chose four paved road sections on Plum Brook Station for our experiment, each of which received little vehicle traffic (<10 vehicles per day; Figure S1). Each section was 1.13 km-long and 3-m wide, and had designated start and end points (i.e., each section was driven in the same direction each time). One section was oriented roughly north to south; the other three sections were oriented southeast to northwest. Also, each section was straight and had little elevation difference; we were able to see the entire length of each section from the start point. Prior to beginning the experiment, we conditioned free-ranging turkey vultures over approximately 14 days to feed at raccoon carcasses (*Procyon lotor*) placed at each section’s end point (see below). All raccoons used in this experiment were salvaged from an Ohio state management program protecting beach-nesting turtles. The carcasses were kept frozen until the night before use.

We gathered field data during 25 days from 1 August to 6 October in 2011 and during 20 days from 25 June to 23 August in 2012. We placed one raccoon carcass in the middle of the road at the end of each road section at approximately 07:30 each day we gathered field data. Carcasses were tethered to a 4.5-kg flat metal weight to prevent them from being dragged from the road. Our general experimental framework consisted of driving a vehicle at one of three constant speeds (30, 60, or 90 kph) directly toward vultures feeding on the raccoon carcasses, and measuring the distance between the approaching truck and the carcass at the time when individual vultures moved to avoid the vehicle (i.e., the flight initiation distance, FID). We used the same route, progressing from road section 1 through 4 (Figure S1), for each replicate of the experiment.

Our approach vehicle was a 2003 Ford F250 pickup truck. To create a consistent visual surface and reduce sun glare off the front of the truck that could have affected vehicle detectability [18], we covered the front of the truck with a flat, dark-green fabric cover measuring 203×98 cm. Only the fabric, the bottom half of the tires, and part of the windshield were visible from the front of the vehicle (Figure S2).

When weather allowed, we made two complete circuits through the four road sections each experimental day; one in the morning and one in the afternoon, separated by at least 4 hr. Because we adhered to the preplanned route, we were reasonably certain that we did not count the same bird twice during a single circuit of the road sections. However, the possibility of pseudoreplication remained, and we took this caveat into consideration with our analyses (see below). Before each circuit, we recorded ambient light conditions (µmol m^−2^ s^−1^), wind speed, and air temperature at the start point of the first road section (Figure S1) and used these metrics as covariates in our models (see below). We recorded ambient light intensity with a Li-Cor (Lincoln, Nebraska, United States of America) LI-250 Light Meter and LI-190SA Quantum Sensor, and wind speed and air temperature with a Kestrel 4500 Pocket Weather Tracker (Nielson-Kellerman, Boothwyn, Pennsylvania, United States of America).

Upon arriving at the start point of a road section, we used a 25× spotting scope to determine whether vultures were present at the carcass location. If no vulture was present, we drove to the next section. If at least one vulture was present, we began our approach by quickly accelerating to one of the three preselected speeds: 30, 60, or 90 kph. Once we reached the desired speed (always within 0.30 km of the start point; Figure S1), we set the cruise control on the truck to maintain constant speed throughout the remainder of the approach. We controlled for the potentially confounding effect of variable starting points [26], [28] by using the same starting distance (1.13 km) for each approach, which was well beyond the FID used by vultures during this experiment. We minimized the difference in engine noise across vehicle speeds by adjusting gears (e.g., the 30 kph approaches were driven in low gear), thus maintaining engine revolutions-per-minute between 1300 and 1600 for all vehicle approach speeds. We cycled through the vehicle speeds in a systematic manner, and conducted 26 approaches for each of the three vehicle speeds over the two years of the study.

Two people participated in each approach, the driver and the observer. The observer focused on the target birds and dropped a bean bag from the vehicle window (File S1) when each bird in the group (ranging in size from 1–9 individuals) initiated an avoidance behavior, defined as any sudden activity, flying or running, that propelled the bird away from the oncoming vehicle [16]. After each vehicle approach, we measured the distances between the dropped bean bags and the raccoon carcass with a Bushnell Yardage Pro 1000 laser range finder (Overland Park, Kansas, United States of America). We corrected each distance for forward momentum at each vehicle speed (File S1) and used the median corrected measured distance for each vulture group as our measurement of FID (see below). The observer also video-recorded approaches using a Canon PowerShot S5IS camera (Canon USA, Inc., Melville, New York, United States of America) mounted within the vehicle just behind the windshield.

### Statistical Analyses

We considered each vehicle approach towards a group of foraging vultures as an experimental unit. One vehicle approach at 60 kph was excluded from our analyses due to a missing record of ambient light intensity. Also, thirteen FIDs of individual vultures >300 m were excluded from calculation of group median FIDs because in these cases vultures were not obviously responding to the vehicle approach [36]. In addition to FID, we calculated time-to-collision (TTC) as *TTC  =  FID/(S × 0.2778)*, where S  =  vehicle approach speed. The constant (0.2778) is the necessary conversion factor when TTC is expressed in s, FID is expressed in m, and S is expressed in kph. In our subsequent analyses, we used the median FID per group (henceforth referred to as FID) and corresponding TTC as response variables, because the data structure within groups suggested that the median was a more accurate measurement of central tendency of responses than the mean.

We first examined potential differences across vehicle approach speeds for the predictor variables: group-size category (1, 2, or >2 individuals), wind speed, ambient light intensity, and air temperature. We normalized group-size category and wind speed via natural logarithm transformation, and ambient light by squaring the value. We then evaluated each variable relative to speed category by using a mixed linear model, Kenward-Rogers adjustment to degrees of freedom, and type III sums of squares. With the exceptions of group-size category and ambient light intensity, a value of zero was possible for the response variable, thus we forced models for wind speed and air temperature through the origin (i.e., removed the intercept). We assessed normality via model residuals, and then examined differences in candidate predictor variables via least squares means (LSM; SAS ver. 8.2, SAS Institute, Cary, North Carolina, United States of America; Table 1).

**Table 1:** Differences in least squares means associated with a mixed linear model comparison of variables measured during responses by free-ranging turkey vultures exposed to the approach of a Ford F250 pickup truck from a standard 1.13-km distance at 30 kph (n** = **25 approaches), 60 kph (n** = **25 approaches, but only 24 considered because of a missing value), or 90 kph (n** = **23 approaches).

Variable*	Approach speed	Estimate†	SE†	DF	*t*	*P*
Air temp (°C)	30 vs. 60	–0.5800	1.3921	70	–0.42	0.6782
	30 vs. 90	–0.3685	1.4220	70	–0.26	0.7963
	60 vs. 90	0.2115	1.4220	70	–0.15	0.8822
Ambient light (µmol m^−2^ s^−1^)	30 vs. 60	8.8848	32.7298	69	0.27	0.7868
	30 vs. 90	39.6501	33.0908	69	1.20	0.2349
	60 vs. 90	30.7653	33.4195	69	0.92	0.3605
Group size	30 vs. 60	–0.0068	0.0824	70	–0.08	0.9345
	30 vs. 90	–0.0911	0.0841	70	–1.08	0.2824
	60 vs. 90	–0.0844	0.0841	70	–1.00	0.3196
Wind speed (kph)	30 vs. 60	0.0030	0.2059	70	0.01	0.9883
	30 vs. 90	0.1957	0.2104	70	0.93	0.3553
	60 vs. 90	0.1927	0.2104	70	0.92	0.3628

* Group size was categorized as 1, 2, or >2 individuals. Group size category and wind speed were transformed via natural logarithm; ambient light intensity was transformed by squaring.

† Because of large values, estimate and SE for ambient light are divided by 10,000.

We then examined potential differences between years for FID and TTC; these data were non-normal, therefore we transformed them via natural logarithm. We evaluated each of the response variables relative to year by again using a mixed linear model, Kenward-Rogers adjustment to degrees of freedom, and type III sums of squares. Here too, a value of zero was possible for both FID and TTC, thus we forced each model through the origin and assessed normality via model residuals and differences in response variables via LSM. We pooled these data upon finding no differences in our response variables between years (2011 approaches n** = **38, mean FID  =  95.3 m, SD  =  62.4 m, Estimate [ln FID]  =  4.3 m; 2012 approaches n** = **35, mean FID  =  106.2 m, SD  =  59.7 m, Estimate  =  4.5 m; LSM estimate  =  –0.1915, df  =  71, *P* **= **0.2287; 2011 mean TTC** = **6.4 s, SD** = **4.4 s, Estimate [ln TTC] ** = **1.8 s; 2012 mean TTC** = **7.0 s, SD** = **3.7 s, Estimate  =  2.0 s; LSM estimate  =  –0.1075, df** = **71, *P* **= **0.3935).

To evaluate differences (*α* **= **0.05) in FID and TTC across vehicle approach speeds and over the two years, we used a mixed linear model with ordinal date as a repeated-measures factor, Kenward-Rogers adjustment to degrees of freedom, and type III sums of squares. For this analysis we used an autoregressive correlation structure because of the possibility that measures taken close in time could contribute to differences in the response variables. As before, we forced each model through the origin. We transformed FID and TTC via natural logarithm and assessed normality via model residuals. For our final model we selected vehicle approach speed as the fixed effect, but also investigated the interactions speed × group-size category, speed × wind speed, speed × ambient light intensity, and speed × air temperature. Although we found no differences in these candidate predictor variables among approach speeds individually (Table 1), we considered the possibility of interaction effects as realistic.

In addition to adjusting our analysis relative to repeated observations by ordinal date, we assumed that any effects of pseudoreplication (i.e., multiple vultures possibly exposed to our approaches repeatedly over short time intervals and over the two years) were reduced by our 4-hour sampling interval and vulture foraging behavior. Specifically, individual variability in timing of foraging, pattern, foraging range (potentially over a 34,000-ha home range) [37], [38], and satiety likely reduced the probability of consistent, repeated vehicle approaches towards the same individual or group on the same road section [39].

No turkey vultures were struck during this experiment, but there were several instances when vultures narrowly avoided our vehicle. To objectively determine what a “near collision” entailed, we used our on-board video recordings of vehicle approaches to estimate the time necessary for turkey vultures to move from the path of the vehicle once a response began. We used 32 recordings that were of sufficient quality to observe vulture reactions unambiguously (generally these were videos of approaches that had low FID values). We viewed videos at one-half speed on a desktop computer and measured the elapsed time between the initiation of flight behavior [16] and the instant when individual vultures cleared the vertical extension of the road edge or flew above the estimated height of the truck. We used the mean value obtained (1.7 s [SE  =  0.9]) as our benchmark for “near collisions”.

### Ethics Statement

The Institutional Animal Care and Use Committee of the United States Department of Agriculture, Animal and Plant Health Inspection Service, Wildlife Services, National Wildlife Research Center approved all procedures used in this study (QA-1855).

## Results

**Figure 1:**
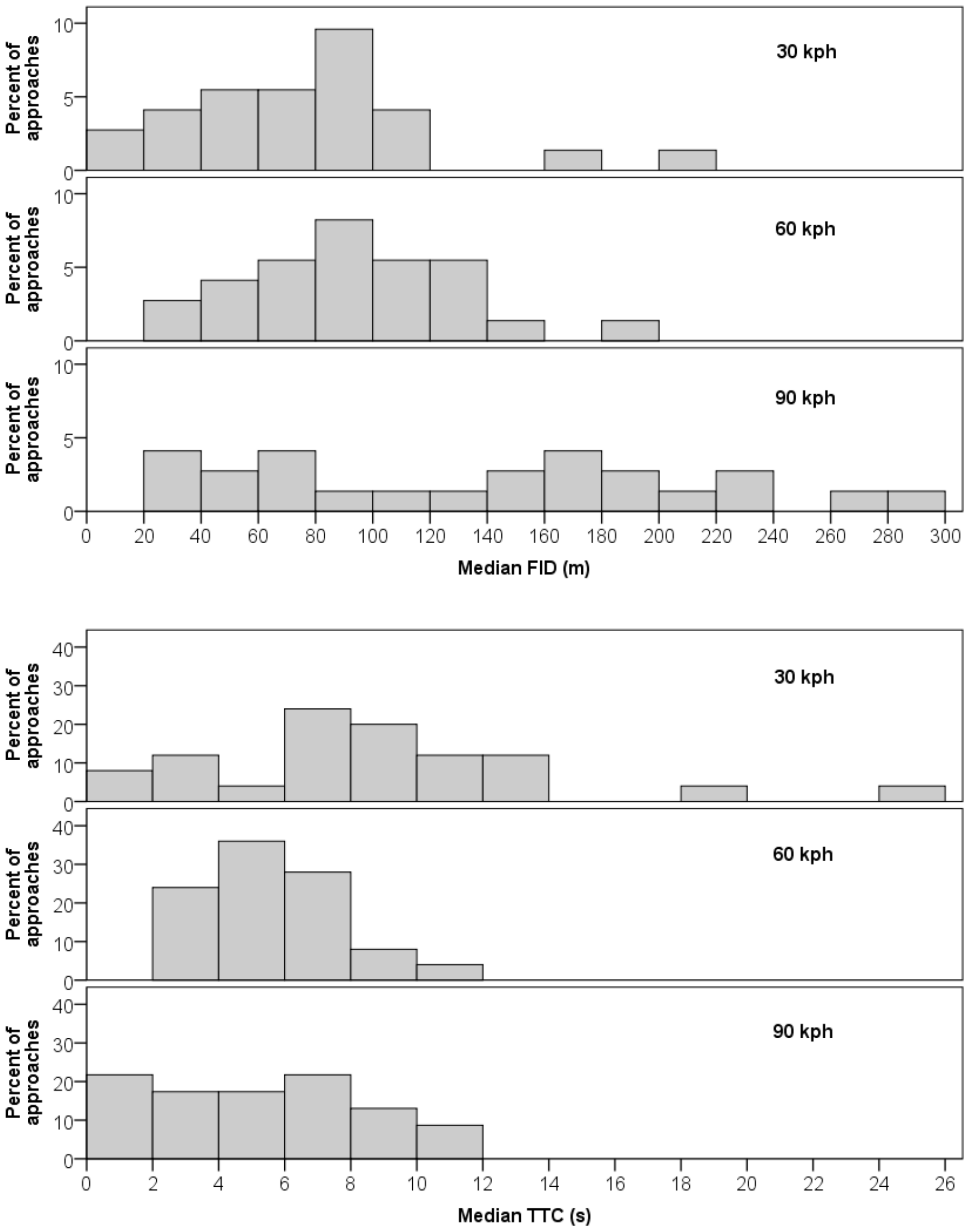
Frequency distributions relative to FID and TTC across 73 vehicle approaches at three vehicle speeds (approaches at 30 kph, n = 25; 60 kph, n = 25; 90 kph, n = 23).

Seventy-two vehicle approaches towards turkey vultures were considered in our model. Of these, 28 approaches involved one vulture, 23 involved two vultures, and 21 involved >2 vultures (group median  =  4; range  =  3–9 individuals). FID of vultures increased by a factor of 1.85 as speed increased from 30 to 90 kph (Table 2). The repeated-measures factor, ordinal date, did not exert a statistically significant effect on FID (Estimated variance  =  –0.1876, residual error  =  0.3876, null model likelihood ratio test: df** = **1, X^2^ **= **0.70, P** = **0.4042). In our final model for FID, vehicle approach speed exerted the only significant effect (Fixed effect F_3/50_ **= **14.5, P <0.0001), despite the various interactions considered (Table 3). Each approach speed exerted a significant effect on FID (Table 3), but we observed significant differences in FID only between 30 and 90 kph (Table 4). Responses between 30 and 60 kph were marginally non-significant (Table 4). Notably, vulture responses varied widely within vehicle speed treatments, especially at 90 kph (Table 2). At 90 kph there was no apparent trend in FID across replicates; birds appeared equally likely to initiate escape behavior at 40 m as at 220 m (Figure 1). The platykurtic distribution of FIDs at 90 kph (kurtosis  =  –1.08) contrasted sharply with the distributions at 30 (kurtosis  =  3.20) and 60 kph (kurtosis  =  0.00), which were less dispersed and had clear modes at 80 to 100 m (Table 2, Figure 1). The distributions of FIDs for all three speeds were positively skewed (skewness  =  1.30, 0.40, and 0.27 for 30, 60, and 90 kph, respectively).

**Table 2:** Flight-initiation distance (FID) and time-to-collision (TTC) for groups of free-ranging turkey vultures exposed to the approach of a Ford F250 pickup truck from a standard 1.13-km distance at 30, 60, and 90 kph.

Response variable	Speed (kph)	N	Mean	SD	CV	Interquartile range
FID (m)	30	25	73.8	44.7	60.6	45.0
	60	25	94.1	36.3	38.6	51.0
	90	23	136.5	79.5	58.2	134.0
TTC (s)	30	25	8.9	5.4	60.6	5.3
	60	25	5.6	2.2	38.6	3.1
	90	23	5.5	3.2	58.2	5.4

See text for description of response metrics.

**Table 3:** Results from a mixed linear model, repeated-measures analysis of flight-initiation distance (FID) for groups of free-ranging turkey vultures exposed to the approach of a Ford F250 pickup truck from a standard 1.13-km distance at 30 kph (n = 25 approaches), 60 kph (n = 25 approaches, but only 24 considered because of a missing value), or 90 kph (n = 23 approaches).

Variable	Approach speed	Estimate	SE	DF‡	*t*	*P*
Approach speed	30	3.4012	1.1840	52.1	2.87	0.0059
Approach speed	60	4.8851	1.1610	52.9	4.21	0.0001
Approach speed	90	4.1416	1.1257	53.7	3.68	0.0005
Ambient light intensity × approach speed	30	0.0003	0.0003	52.9	0.93	0.3543
Ambient light intensity × approach speed	60	–0.0005	0.0004	53.2	–1.30	0.1978
Ambient light intensity × approach speed	90	–0.0002	0.0003	54.0	–0.50	0.6158
Air temperature × approach speed	30	0.0296	0.0420	51.2	0.70	0.4842
Air temperature × approach speed	60	0.0045	0.0444	52.1	0.10	0.9188
Air temperature × approach speed	90	0.0302	0.0383	53.1	0.79	0.4341
Approach speed × group size category 1*	30	–0.0468	0.3728	52.5	–0.13	0.9006
Approach speed × group size category 2*	30	–0.3142	0.4153	52.5	–0.76	0.4527
Approach speed × group size category 3*	30	0.0000†	−	−	−	−
Approach speed × group size category 1*	60	0.1680	0.3335	54.0	0.50	0.6165
Approach speed × group size category 2*	60	0.0333	0.3998	49.3	0.08	0.9340
Approach speed × group size category 3*	60	0.0000†	−	−	−	−
Approach speed × group size category 1*	90	–0.4303	0.3927	54.0	–1.10	0.2781
Approach speed × group size category 2*	90	–0.0798	0.3481	53.8	–0.23	0.8195
Approach speed × group size category 3*	90	0.0000†	−	−	−	−
Wind speed × approach speed	30	–0.0613	0.0407	53.4	–1.51	0.1375
Wind speed × approach speed	60	–0.0014	0.0363	47.1	–0.04	0.9684
Wind speed × approach speed	90	0.0296	0.0594	54.0	0.50	0.6200

Ordinal date served as the repeated-measures factor and FID was transformed via natural logarithm to meet requirements for normality. Approach speed served as the fixed effect.

* Group size category (1, 2, 3** = **1, 2, or >2 vultures per approach, respectively) entered only in the interaction with approach speed.

† We selected the NOINT (no intercept) option for Proc Mixed used because of the realistic possibility of no response (i.e., FID** = **0) to vehicle approach. Dashes indicate inestimable effects relative to DF for the interaction of each approach speed category and the third group size category.

‡ DF represent Kenward-Rogers approximation of degrees of freedom (SAS/STAT Users Guide Version 8).

**Table 4:** Differences in least squares means associated with a mixed linear model, repeated-measures analysis of flight-initiation distance (FID) and time-to-collision (TTC) for groups of free-ranging turkey vultures exposed to the approach of a Ford F250 pickup truck from a standard 1.13-km distance at 30 kph (n = 25 approaches), 60 kph (n = 25 approaches, but only 24 considered because of a missing value), or 90 kph (n = 23 approaches).

Response variable	Approach speeds	Estimate	SE	DF	*t*	*P*
FID	30 vs. 60	–0.3795	0.1990	50.6	–1.91	0.0623
	30 vs. 90	–0.6046	0.1992	53.9	–3.04	0.0037
	60 vs. 90	–0.2251	0.2056	53.8	–1.10	0.2783
TTC	30 vs. 60	0.2939	0.1608	49.4	1.83	0.0736
	30 vs. 90	0.4139	0.1617	54.0	2.56	0.0133
	60 vs. 90	0.1201	0.1666	53.7	0.72	0.4743

Ordinal date served as the repeated-measures factor and FID was transformed via natural logarithm to meet requirements for normality. Approach speed served as the fixed effect. See Table 3 for specific details on the mixed linear model analysis.

TTC decreased by a factor of 0.62 with approach speeds from 30 to 90 kph, and because of the relationship of this metric to FID, variation across approach speeds was similar to that observed for FID (Table 2). As with FID, vehicle speed was the only significant predictor of TTC in our final model (Fixed effect F_3/50.2_ **= **3.47, P** = **0.0228; Table 5). Although we again observed significant difference between responses at 30 and 90 kph (Table 4), we attribute the lack of effect on TTC between 30 and 60 kph, in part, to the wide variance in responses within vehicle speed treatments (Table 2, Figure 1). Although mean TTCs were similar at 60 and 90 kph (Table 2), more near collisions occurred at 90 kph—there were four TTCs ≤1.7 s for approaches at 90 kph (17%), no such TTCs for 60 kph, and one for 30 kph (4%).

**Table 5:** Results from a mixed linear model, repeated-measures analysis of time-to-collision (TTC) for groups of free-ranging turkey vultures exposed to the approach of a Ford F250 pickup truck from a standard 1.13-km distance at 30 kph (n = 25 approaches), 60 kph (n = 25 approaches, but only 24 considered because of a missing value), or 90 kph (n = 23 approaches).

Variable	Approach speed	Estimate	SE	DF‡	*t*	*P*
Approach speed	30	1.5906	0.9641	52.5	1.65	0.1049
Approach speed	60	2.1511	0.9444	53.2	2.28	0.0268
Approach speed	90	1.2254	0.9141	53.7	1.34	0.1857
Ambient light intensity × approach speed	30	0.0003	0.0003	53.2	0.97	0.3382
Ambient light intensity × approach speed	60	–0.0004	0.0003	53.3	–1.38	0.1722
Ambient light intensity × approach speed	90	–0.0001	0.0003	54.0	–0.52	0.6031
Air temperature × approach speed	30	0.0226	0.0342	51.8	0.66	0.5114
Air temperature × approach speed	60	0.0055	0.0361	52.7	0.15	0.8787
Air temperature × approach speed	90	0.0255	0.0311	53.1	0.82	0.4153
Approach speed × group size category 1*	30	–0.0602	0.3035	53.2	–0.20	0.8436
Approach speed × group size category 2*	30	–0.2532	0.3381	53.1	–0.75	0.4572
Approach speed × group size category 3*	30	0.0000†	−	−	−	−
Approach speed × group size category 1*	60	0.1415	0.2708	54.0	0.52	0.6035
Approach speed × group size category 2*	60	0.0203	0.3264	50.4	0.06	0.9506
Approach speed × group size category 3*	60	0.0000†	−	−	−	−
Approach speed × group size category 1*	90	–0.3139	0.3187	54.0	–0.98	0.3291
Approach speed × group size category 2*	90	–0.0576	0.2827	53.8	–0.20	0.8394
Approach speed × group size category 3*	90	0.0000†	−	−	−	−
Wind speed × approach speed	30	–0.0486	0.0330	53.5	–1.47	0.1474
Wind speed × approach speed	60	–0.0016	0.0296	47.8	–0.06	0.9563
Wind speed × approach speed	90	0.0240	0.0482	54.0	0.50	0.6198

Ordinal date served as the repeated-measures factor and TTC was transformed via natural logarithm to meet requirements for normality. Approach speed served as the fixed effect.

* Group size category (1, 2, 3** = **1, 2, or >2 vultures per approach, respectively) entered only in the interaction with approach speed.

† We selected the NOINT (no intercept) option for Proc Mixed used because of the realistic possibility of no response (i.e., FID  =  0) to vehicle approach. Dashes indicate inestimable effects relative to DF for the interaction of each approach speed category and the third group size category.

‡ DF represent Kenward-Rogers approximation of degrees of freedom (SAS/STAT Users Guide Version 8).

## Discussion

Responses of turkey vultures to the oncoming vehicle varied by vehicle speed, both in terms of the mean response and the distribution of responses. The wide range of FIDs (and by extension, TTCs) that we observed within speed treatments, especially at 90 kph, reflects substantial variation in response to vehicles within or among individuals of a given species [21], [39]–[41]. In a meta-analysis of factors that influence fear in animals (as measured by FID), Stankowich and Blumstein [42] found that predator traits (e.g., speed, size, and directness of approach) often had the most consistent influence on FID. However, in our study the analogous factors (traits inherent to the approach vehicle) were standardized across replicates within vehicle speed treatments, and thus should not have influenced our results. Instead, we suspect that differences in escape responses within speed treatments could have been based, in part, on hunger level [43], experience with vehicles [7], and variation in tolerance to disturbance inherent among individuals [41].

Potential limitations on optimal escape behaviors imposed by increased vehicle speeds might be illuminated by considering the proximate cues animals use to decide when to initiate flight responses to avoid vehicles and other threats [25], [44]. Such decisions can be based on a fixed FID (i.e., a zone of awareness) [26], a ratio of FID to alert distance [27], or an estimate of TTC. For instance, Wang and Frost [45] empirically demonstrated that rock pigeons (*Columba livia*) possess looming-sensitive neurons that selectively respond to objects on a direct collision course (projected on a computer-generated display) and stimulate escape responses at a consistent TTC, regardless of object size or speed. These looming-sensitive neurons are thought to encode the optical variable tau [30], [46], which is calculated as the angle of the object subtended on the retina (a function of object size and distance) divided by the rate of angular expansion of that object as it approaches. However, the Wang and Frost [45] experiment was limited to (virtual) objects approaching at a maximum of 27 kph, corresponding roughly to the speed of natural predators. Because tau estimates TTC irrespective of the oncoming object’s size and speed, to elicit an escape response when an object approaches at a greater speed (e.g., 90 kph), tau neurons would need to begin firing when the oncoming object projects a much smaller retinal image (i.e., when it is further away). Gibson [30], [47] demonstrated that estimation of object size by humans is more variable for far-away objects than for those near to the observer. If the same is true for birds, the result could lead to greater error (i.e., greater variance) in estimating TTC for fast vehicles than for slower ones, as suggested by our results for turkey vultures (Figure 1).

Estimation of TTC in response to vehicle approach could be influenced by factors other than vehicle speed and its effects on the looming image, including vehicle size, color, and other aspects of its appearance. Also, in many cases vehicles potentially intersecting the movement paths of birds might not be viewed “head-on”, and thus not provide an image that expands symmetrically on the retina, a condition necessary for images to loom in the traditional sense [45]. Therefore, the orientation and trajectory of the vehicle’s image on the retina might be important for the accurate estimation of TTC and subsequent initiation of successful avoidance responses. It is conceivable that birds may modify their behavior, including orientation of their heads in relation to the approaching vehicle, to take advantage of the looming effect. Alternatively, other aspects of optic flow [30], including the integration of multiple cues [48], might be used in some cases for accurate timing of escape behaviors.

Irrespective of the proximate cues employed, the behavioral mechanisms of object avoidance are inherently linked to a fitness strategy that will vary with perception of risk and energy status. More specifically, theory predicts that animals will remain in place (i.e., they will continue to feed, rest, etc.) until it is more optimal to leave; in most cases animals will not flee immediately upon detecting a threat [23], [32], [44]. This could be especially true for approaching vehicles. For example, because almost all vultures in our study were adults (as indicated by color of the head [49]), many individuals likely had experience foraging along roads [34], [37], and thus might have expected the approaching vehicle to travel along the road in a predictable manner until it reached the point of potential collision. In such a scenario where vehicles are clearly differentiated from natural predators (which can quickly change speeds or movement paths and thus require more careful monitoring [50]), the most efficient behavior might be for individuals to continue feeding until late in the vehicle approach, and then initiate an avoidance response. It is not difficult to envision how this type of behavior might leave individuals vulnerable to collision with especially fast-moving vehicles. Even so, the high variability in responses we observed suggests that other factors unmeasured in this experiment (see below) may be interacting to influence the timing of these avoidance behaviors.

Although turkey vultures in our study increased FID with vehicle speed, the difference in response distributions among speed treatments (i.e., more dispersion at 90 kph) suggests that escape rules used to avoid vehicles by turkey vultures are not equally effective across all vehicle speeds (Figure 1). For example, most near collisions (TTC ≤1.7 s) occurred during 90 kph vehicle approaches. Our findings therefore suggest that turkey vultures successfully use escape rules only up to a threshold speed, and that vehicle avoidance behaviors may not be well tuned to vehicles approaching at these high speeds, possibly due to behavioral and physiological limitations imposed by cognitive processing of visual information. Had we approached vultures at vehicle speeds ≥90 kph, we suspect that collisions would have been even more likely.

We do not mean to imply that all bird-vehicle collisions (BVCs) are caused by miscalculations involving the speed of oncoming vehicles. Other factors such as distractions or risk-taking behavior related to hunger [43] likely contribute substantially to the prevalence of BVCs (unpublished data). However, if birds rely on innate antipredator behaviors in response to non-predator, approaching threats [18], [19], [23], [24], and these behaviors are based, at least in part, on decision rules incorporating distance or estimation of TTC via the mechanisms discussed above [45], then our results suggest some individuals may be at a high risk of collision when confronted with particularly fast-approaching vehicles. Especially considering that aircraft often travel at several hundred kph within the typical flight altitudes of vultures and other birds [35], [51], vehicle speed could be a major contributor to bird-aircraft collisions that involve a wide range of taxa.

BVCs on roads often increase as posted speed limits increase [52]. Further, Erritzoe et al. [1] suggested that few BVCs occur below 40 kph, and that especially fast vehicles might be responsible for most collisions. These studies, in combination with our data, suggest that reducing vehicle speeds by lowering posted speed limits, installing speed bumps or other physical barriers, or other means could reduce BVCs. For example, only one of the 50 vehicle approaches in our study ≤60 kph resulted in a near collision. Although reduced speed limits are sometimes used to reduce the likelihood of human injuries in areas where ungulate-car collisions are common [8], this approach is considered less often as a management option in the context of conservation. As the role of vehicle speed in contributing to BVCs becomes clearer [33], we suggest that lowering speed limits should be considered where this type of approach is practical, such as in wildlife parks and reserves, and other areas inhabited by birds of conservation concern. Reducing vehicle speeds might be especially important for smaller bird species, which generally have lesser FIDs [42], [53], and thus might be at even greater risk from fast vehicles than vultures and other large species.

We also suggest that more emphasis should be placed on research aimed at better understanding avian detection and response to high-speed vehicles (both automobiles and aircraft), and research that explores how to enhance avoidance behaviors using lights, paint schemes, or other onboard effects [16], [18], [19], [54]. Research focused on (1) elucidating behavioral rules used by various species to initiate avoidance responses to vehicles, (2) determining the threshold speeds at which responses become ineffective, and (3) manipulating avian detection of vehicle approach and perception of vehicle speed (i.e., enhancing the looming response [55]), would be especially useful. Because bird responses to oncoming threats can vary considerably among species [18], [53], [56], we encourage a multi-species approach, with priority on species of conservation concern and those that are involved in the most damaging aircraft collisions [13]. Further, we encourage research examining bird responses to even faster vehicles, although such investigations will likely require an innovative combination of field and laboratory studies.

## Supporting Information

Figure S1 **Aerial image of NASA Plum Brook Station in north-central Ohio, USA, and locations of four road sections (on the perimeter of the property) where vehicle approaches were made towards turkey vultures feeding along roads.** The diagram at right represents measurements for each road section.
(TIF)

Figure S2 **Road-level view of Ford F-250 with fabric screen mounted to cover front of truck to reduce unintended glare.**
(TIF)

File S1 **Supplementary field methods.**
(DOCX)
