# Supplementary figures and images for: Effects of Vehicle Speed on Flight Initiation by Turkey Vultures: Implications for Bird-Vehicle Collisions
Source: PLoS One. 2014 Feb 4;9(2):e87944. doi: 10.1371/journal.pone.0087944 (PMC3913678; doi:10.1371/journal.pone.0087944)

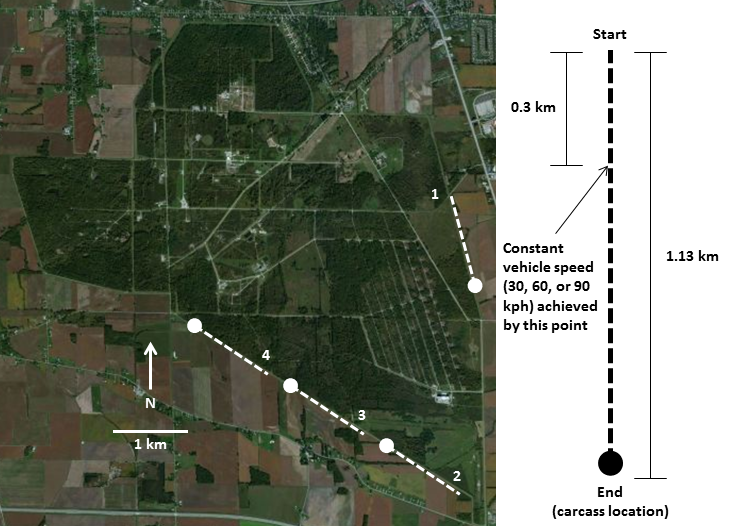

Supplement: Figure S1 — Aerial image of NASA Plum Brook Station in north-central Ohio, USA, and locations of four road sections (on the perimeter of the property) where vehicle approaches were made towards turkey vultures feeding along roads. The diagram at right represents measurements for each road section. (TIF) [file pone.0087944.s001.tif]

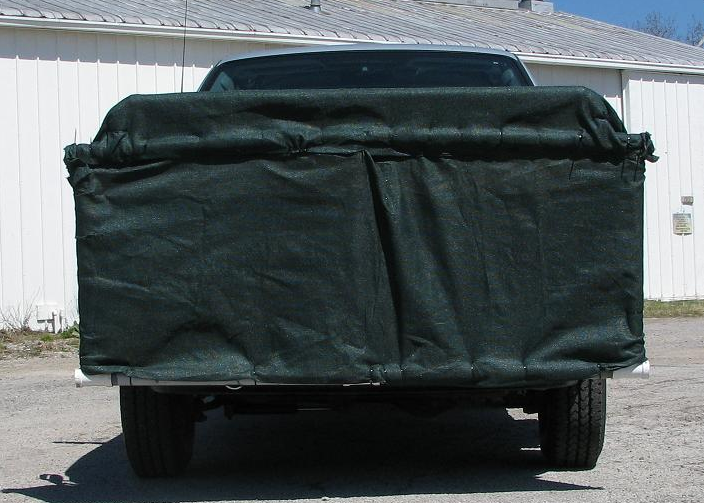

Supplement: Figure S2 — Road-level view of Ford F-250 with fabric screen mounted to cover front of truck to reduce unintended glare. (TIF) [file pone.0087944.s002.tif]
