# Supplementary material for: Effects of Vehicle Speed on Flight Initiation by Turkey Vultures: Implications for Bird-Vehicle Collisions
Source: PLoS One. 2014 Feb 4;9(2):e87944. doi: 10.1371/journal.pone.0087944 (PMC3913678; doi:10.1371/journal.pone.0087944)
Supplement: File S1 — Supplementary field methods. (DOCX) [file pone.0087944.s003.docx]

**File S1.** Supplementary field methods

**Correcting measurements of FID for forward momentum**

We used wooden stakes to visually mark five points on the margins of a straight road at NASA Plum Brook Station at 100 m intervals. We then drove past these markers at each of our three vehicle approach speeds and dropped bean bags from the vehicle window at the points when the vehicle was adjacent to the markers. Using a meter tape, we measured the distance from the dropped bean bags to the stakes to the nearest m. Results are presented in the table below.

| Replicate | 30 kph | 60 kph | 90 kph |
| --- | --- | --- | --- |
| 1 | 3 | 8 | 16 |
| 2 | 2 | 8 | 16 |
| 3 | 3 | 8 | 14 |
| 4 | 2 | 7 | 15 |
| 5 | 3 | 9 | 15 |
| Mean | 2.6 | 8.0 | 15.2 |

Based on these results, we added 3 m, 8 m, and 15 m to each of the individual vulture responses at 30 kph, 60 kph, and 90 kph, respectively, before calculating the median response for each vulture group (experimental unit).

**Testing for differences in reaction time of observers**

One of two individuals served as observer, dropping bean bags to measure FID, during 92% of all vehicle approaches (41 approaches for TWS; 26 for BFB). To determine whether observers had different reaction times which may have influenced measurements of FID, we conducted a two-sample T-Test with observer as the independent variable and FID as the dependent variable. The mean FIDs did not differ (T = -0.72, P = 0.474), suggesting that reaction times were similar between observers.
